# The Salience of Complex Words and Their Parts: Which Comes First?

**DOI:** 10.3389/fpsyg.2016.01778

**Published:** 2016-11-21

**Authors:** Hélène Giraudo, Serena Dal Maso

**Affiliations:** ^1^CLLE, University of Toulouse, CNRSToulouse, France; ^2^Department of Cultures and Civilizations, University of VeronaVerona, Italy

**Keywords:** morphological salience, visual word recognition, morphological processing, masked priming, lexical access

## Abstract

This paper deals with the impact of the salience of complex words and their constituent parts on lexical access. While almost 40 years of psycholinguistic studies have focused on the relevance of morphological structure for word recognition, little attention has been devoted to the relationship between the word as a whole unit and its constituent morphemes. Depending on the theoretical approach adopted, complex words have been seen either in the light of their paradigmatic environment (i.e., from a paradigmatic view), or in terms of their internal structure (i.e., from a syntagmatic view). These two competing views have strongly determined the choice of experimental factors manipulated in studies on morphological processing (mainly different lexical frequencies, word/non-word structure, and morphological family size). Moreover, work on various kinds of more or less segmentable items (from genuinely morphologically complex words like *hunter* to words exhibiting only a surface morphological structure like *corner* and irregular forms like *thieves*) has given rise to two competing hypotheses on the cognitive role of morphology. The first hypothesis claims that morphology organizes whole words into morphological families and series, while the second sets morphology at a pre-lexical level, with morphemes standing as access units to the mental lexicon. The present paper examines more deeply the notion of morphological salience and its implications for theories and models of morphological processing.

## What is Salient in Morphological Processing?

In linguistics, the semiotic notion of salience has been applied to inflectional and derivational morphology from the 1980s onward, mainly in the framework of ‘Natural Morphology’ (NM; e.g., Dressler et al., 1987). In this approach, the idea of morphological salience refers to the relative importance or prominence of a morpheme (stem or affix) in a morphologically complex word, the underlying assumption being that the salience of morphological components drives the mechanisms underlying complex word processing as well as storage and lexical organization. More recently, in the domain of language acquisition, Goldschneider and DeKeyser (2001) defined morphological salience as referring to “how easy it is to hear or perceive a given structure” (p. 22).

In the Natural Morphology (henceforth: NM) approach, salience is one of the factors that contribute to the ‘naturalness’ of a linguistic item or structure, which in turn determines how easily it can be processed by the human brain (Dressler et al., 1987, p. 11). Thus, NM theory explicitly defines naturalness on psychological grounds and makes particular reference to cognitive limitations on perception and processing (e.g., on memory, information recall, and selective attention). According to Natural Morphologists, psycholinguistic factors do not directly determine linguistic structures, but they limit the choice of available linguistic (in our case morphological) techniques, favoring the ones that are cognitively less demanding and disfavoring the more cognitively demanding ones. In a way, psycholinguistic factors ‘constrain’ the possibilities of languages.

Two kinds of factors are supposed to determine the salience of the components of a morphologically complex word, thereby affecting the recognition of its morphological structure. The first group of factors relates to the strength of the mental representation of the whole complex word and its components, which is thought to be modulated by the following variables: (i) (token and type) frequency; (ii) numerosity (i.e., the number of distinct words with which a suffix occurs, cf. Burani and Thornton, 2003); (iii) productivity. Intuitively, the more frequently a form is heard and processed, the stronger a mental representation it has and the easier it is to recognize. The second group of factors relates to more formal characteristics of morphemes and involves a wide range of features, in particular: (i) their size and phonological features (e.g., stress); (ii) their position within the complex word (i.e., initial, final, or internal); (iii) their formal (in)variance (i.e., the less an item varies in a paradigm, the more recognizable it is); (iv) the morphotactic transparency of the complex word they are embedded in; (v) their formal distinctness [i.e., if a morphological component is salient, its form is distinct paradigmatically both with respect to forms of the same morphological family or paradigm and with regard to forms which are formally similar but semantically unrelated (i.e., the orthographic neighborhood, Andrews, 1989, 1992)].

More broadly, the salience of a morphological item may also be influenced by semantic and functional properties, such as consistency (a formal component is recognized more easily if it always occurs with the same meaning or function) and morpho-semantic transparency (the constituents fully contribute to the meaning of the complex word, see Plag, 2003). The present paper will discuss the extent to which experimental psycholinguistic studies have confirmed the psychological plausibility of the notion of salience and its effects in word processing and lexical organization.

## The Whole-Word and Decompositional Perspectives

Differing stances on the nature and role of morphology within the mental lexicon have led to two opposite hypotheses about processing: either morphemic representations stand as access units to word representations, or word representations organize the mental lexicon into morphological families. According to the first view, which is often referred to as the “decompositional view,” the morphemic units correspond to concrete pieces of words (i.e., stems and affixes), coded at a sublexical level and processing complex words implies passing through a decomposition mechanism that strips off the affix in order to isolate the stem, so that the morphemic nature of the remaining letters can be checked by the system. Access to word representations (i.e., word forms coded in the orthographic lexicon) thus operates via the pre-activation of their constituent morphemes. This mechanism is exemplified in the interactive activation model developed by Taft (1994), a model instantiating the decompositional view of morphology by integrating sublexical morphemic representations as access units.

According to the second view, called the “whole-word perspective,” morphology is represented at the interface of word and semantic representations and derives from lexemes as introduced by Aronoff (1994), i.e., lexeme units are coded at a *morphomic* level and have the function of organizing the lexicon in terms of morphological families. In processing terms, the recognition of any complex word initially triggers the activation of all word forms that can match with it, and a competition is then engaged between the pre-activated forms until the right lexical representation reaches its recognition threshold (determined by its surface frequency). During this competition phase, competitors send positive activation to their respective base lexemes, which send positive activation back to them. According to this account, exemplified in the supralexical model of Giraudo and Grainger (2000), complex words are not “decomposed” following the procedure described by the sublexical/decompositional account, but are able to trigger the activation of their constituent morphemes.

Both sublexical and supralexical approaches to morphological processing integrate a morphological level of processing, however, they differ with respect to the location of morphological units within the architecture of the mental lexicon, as well as the content of these units, both of which properties define their role of such units in word processing. According to the sublexical view, morphemic units stand as access units, situated between the letter/syllable level and the word level: consequently, these units can only correspond to concrete letter clusters that constitute words (i.e., bound stems, free stems, and affixes) and are insensitive to any semantic characteristics of words (i.e., transparent vs. opaque) or to their lexical environment (in terms of orthographic neighborhood or family size). On the other hand, the supralexical view situates morphological units above the word-forms and before the semantic units. These intermediate units are supposed to be abstract enough to tolerate form variations induced by the processes of derivation and inflection. This implies that a morphemic unit does not need to exist in the real world in order to be coded in long-term memory, but that its existence/emergence depends on the interactions between the word-form and the semantic levels; it also implies that all morphemes of a given language are not necessarily represented within the mental lexicon: unknown words, neologisms, hapaxes, and nonce words are not necessarily connected with morphemic units.

However, determining which factors are involved in lexical access and which factors influence the organization of the mental lexicon are issues that have not been sufficiently explored so far, although they are highly relevant to lexical modeling. We suggest that it is crucial to keep these issues apart: the factors driving the early stages of processing are likely to be different from those coded in long-term memory. In our view, observing sensitivity to the internal structure of complex words can be interpreted as reflecting a central role of morphemes in lexical access, but the factors influencing lexical access (e.g., lexical frequency) are likely to be different from those organizing the mental lexicon properly (e.g., morphological family size).

## Evidence Taken to Support the Decompositional Approach

Numerous psycholinguistic studies have addressed the issue of morphological processing during word recognition. Using the lexical decision task (in which participants must make a decision about whether combinations of letters are words or not), these studies explore the factors influencing the processing of complex words as well as their internal structure. Among these factors, surface frequency (equivalent to token or lemma frequencies) and base frequency (the token or lemma frequency of a root), which measure the statistical occurrence of complex words, have been extensively studied in languages for which lexical databases are available (e.g., Taft, 1979, 2004; Burani et al., 1984; Burani and Caramazza, 1987; Colé et al., 1989, 1997; Baayen et al., 1997; Bertram et al., 1999, 2000a; Burani and Thornton, 2003; Ford et al., 2010; Xu and Taft, 2015). These studies show, among other findings, that when two words are matched in terms of surface frequency (SF), reaction times depend on their base frequency (BF), with high BF words being recognized faster than low BF words. The fact that recognition latencies for complex words depend on base frequencies has been taken as evidence that readers are sensitive to morphological structure and that a cognitive component of word processing is related to the perceptual salience of both the whole word and its morphemic structure. These data gave rise to the decompositional hypothesis as reflecting the automatic processing of morphemes by the cognitive system.

Many studies have lent further support to the decompositional approach to complex word recognition, using priming, and, more recently, masked priming (Forster and Davis, 1984). In masked priming, a prime word is presented for a very short duration (under 60 ms) and is masked by a backward font (usually a string of hash marks), before a target word on which subjects have to perform a lexical decision task is presented. Because this duration does not allow the participants to identify the prime consciously, this paradigm has the advantage of examining very early automatic processes of lexical access as well as non-strategic responses based on the relationships shared by the prime-target pairs (see Forster, 1999, for a review). From the seminal repetition priming study conducted by Stanners et al. (1979) to the most recent studies investigating the brain correlates of masked priming (e.g., Morris et al., 2013), morphological priming effects have been extensively studied and have systematically revealed strong facilitation. Morphological effects (i.e., a morphologically complex prime like *hunter* facilitating the recognition of its morphologically related target *hunt*) differing significantly from formal (e.g., *hungry-hunt*) and meaning relationships (e.g., *pursuit-hunt*), have led the authors to conclude that independent morphological representations are coded somewhere within the mental lexicon in a similar way to orthographic, phonological, and semantic representations. Therefore, until the beginning of the 21st century, experimental studies considered morphological effects to result from systematic form-meaning correlations.

However, between 2000 and 2005, many masked priming studies started to focus exclusively on formal aspects of complex words, that is on their so-called ‘morphological surface structure’ (e.g., Rastle et al., 2004, p. 1091) in order to examine whether processing is decompositional or holistic. The underlying hypothesis was that if significant priming effects can emerge only from the surface structure of words (i.e., from form only), whether morphologically complex or not, then morphology is not coded within the lexicon but rather in its access routes. It is important to highlight here that this approach to morphological complexity, which considers only the surface forms of words, is based on the assumption that morphology can be emptied of its meaning component. Consequently, according to this view separating morphology from semantics, morphological regularities within languages exclusively increase the ‘surface’ salience of morphemes, the aim being to guide pre-lexical processes.

While the priming study carried out by Rastle et al. (2000) historically defines the starting point of this series of masked priming studies, the most striking ones were conducted, respectively, for French by Longtin et al. (2003) and for English by Rastle et al. (2004). Both manipulated word pairs involving primes with morphologically pseudo-complex surface forms (e.g., the English word *corner*, which cannot be decomposed into the morphemes *corn-* and *-er*). Using the masked priming paradigm, it was shown that pseudo-derived word primes (e.g., *corner*) as well as pseudo-derived non-word primes (i.e., non-words composed of two existing morphemes such as *corning*) were able to produce significant priming effects on the recognition times of their pseudo-base (e.g., *corn*). Moreover, the studies found both the quality and the magnitude of these priming effects to be comparable to the priming effects produced by genuinely derived words (e.g., *banker-bank*). Finally, the systematic use of orthographic control primes (i.e., morphologically simple forms whose onset alone mimics a stem morpheme, such as *brothel*, whose ending *-el* never functions as a suffix in English) in these studies showed that these surface morphological effects could not be assimilated to mere formal overlap. Consequently, these effects were taken to result exclusively from the surface morphological structure of the primes.

Further masked priming studies have tested the effect of pseudo-derived non-words primes, and systematically found facilitation effects, lending strong support to the notion of an early mechanism of form decomposition that is applied to all morphologically structured stimuli (McCormick et al., 2009; Morris et al., 2013; Beyersmann et al., 2014; Crepaldi et al., 2016). In general, the logic behind such studies is that since non-words are not supposed to have lexical representation(s), any masked priming effect obtained must reflect activation of sublexical units, i.e., morphemes. Thus, in a recent review, Amenta and Crepaldi (2012) claimed that “morphological effects in non-words exclude the possibility that morphological information only comes into play after lexical identification” (p. 9), given that “it is clear that non-words with a morphological structure are analyzed in terms of their morphemes, thus questioning seriously any theory that suggests morphological processing to kick off upon lexical identification” (p. 7). For example, Longtin and Meunier (2005) used pseudo-derived pseudo-words to test the robustness of early morphological decomposition. In their masked priming study, non-existent possible words created from two existing morphemes (for instance, the base *sport*- combined with the suffix *-ation* to produce *sport-ation*) were used as primes. The data revealed that pseudo-word primes like *sportation* facilitate the recognition of their base (e.g., *sport*) with no difference from the facilitation effects obtained using transparent primes (e.g., *sportif*, which is a licit and semantically transparent derivation from the base *sport*).

Studies showing masked morphological priming effects without semantic relationships have been broadly replicated in various languages (Spanish: Sánchez-Casas et al., 2003; German and French: Diependaele et al., 2005, 2009; French: Giraudo and Voga, 2013; Arabic: Boudelaa and Marslen-Wilson, 2004a,b, 2005; English: Lavric et al., 2007; Marslen-Wilson et al., 2008; Feldman et al., 2009, 2015; McCormick et al., 2009; Lehtonen et al., 2011^1^; Finnish: Järvikivi and Pyykkönen, 2011 and Russian: Kazanina et al., 2008; Kazanina, 2011).

All these studies led the authors to conclude that the morphological decomposition mechanism transcends stimuli and languages. A review by Rastle and Davis (2008) clearly set out that “morphological decomposition is a process that is applied to all morphologically structured stimuli, irrespective of their lexical, semantic or syntactic characteristics” (p. 949). Further evidence in support of this view was provided by a study by McCormick et al. (2008), who manipulated a particular category of derived stimuli that cannot be segmented perfectly into their morphemic components (e.g., *dropper-drop*, in which there is a duplicated consonant) in order to test the flexibility of the morpho-orthographic segmentation process described by decompositional models. Once again, their results were interpreted as demonstrating the robustness of the decomposition process in the case of various orthographic alterations in semantically related (e.g., *adorable-adore*) as well as unrelated prime-target pairs (e.g., *fetish-fete*).

## Objections to the Decompositional Approach

The results reported in the previous section have largely been taken to support a decompositional approach. However, in our view, there are also studies that are inconsistent with this interpretation.

Some masked priming studies have indeed demonstrated very early semantic influences in word recognition. Feldman et al. (2009) matched affixes across semantically transparent and opaque related (and unrelated) prime-target pairs and increased the proportion of identical prime-target filler pairs (e.g., *artist-artist*) in order to enhance semantic facilitation (e.g., Bodner and Masson, 2003). They found that morphological facilitation was significantly greater for semantically transparent pairs (e.g., *coolant-cool*) than for opaque pairs (e.g., *rampant-ramp*). Giraudo and Voga (2013) manipulated prefixed words (e.g., *prénom* ‘name’) and non-words (e.g., *dénom* = *dé-* + *-nom*) in French. They showed that when compared to unrelated primes, both prefixed words and prefixed non-words facilitate target recognition. However, when compared to an orthographic non-word condition (e.g., *danom*), pseudoprefixed primes do not differ from orthographic primes, suggesting a strong formal component in surface morphological priming with semantics. Finally, Feldman et al. (2015) tracked the time course of processing of the interaction between form and meaning using different prime exposure durations (increasing from 34 to 100 ms). They observed that the time course of facilitation varies for similar forms with and without semantic similarity, the transparency effect being evident even at an SOA of 34 ms (Experiment 3).

Other studies have explored the interaction of frequency effects with paradigmatic factors such as affix type and suffix productivity. In a series of lexical decision task experiments, Colé et al. (1989) and later Beauvillain (1996) with eye-movement recordings, showed that while suffixed word recognition in French is sensitive to the manipulation of both types of frequencies (SF and BF), prefixed word recognition is affected only by SF. The authors suggested that this asymmetry could simply reflect the left-to-right direction of the reading process, but studies using other paradigms such as masked priming refuted this physical explanation (e.g., Giraudo and Grainger, 2003). Moreover, Bertram et al. (2000b) discovered that BF effects in Dutch emerge only for words with a very productive suffix. This interaction between BF and affix productivity was replicated for English by Ford et al. (2010), who found that this effect occurs independently of the morphological family size effect, suggesting the occurrence of both holistic and compositional effects during complex word recognition. Only three studies have so far investigated frequency effects using masked priming, and the results have been inconsistent. Giraudo and Grainger (2000) manipulated the SF of derivatives used as primes for the same target (High SF *amitié* - *ami* ‘friendship-friend’; Low SF *amiable - ami* ‘friendly friend’) and found an interaction between priming effects and the prime SF (Experiment1), but no effect for the BF. Experiments 1 and 3 demonstrated that the SF of morphological primes affects the degree of morphological priming: high SF derived primes show significant facilitation relative to orthographic control primes (e.g., *amidon - ami* ‘starch-friend’), whereas low SF primes do not. The results of Experiment 4 revealed, by contrast, that BF does not influence the size of morphological priming on free root targets. Suffixed word primes facilitate the processing of free root targets with low and high BF. These data support the relevance of the whole word form (as reflected by SF) over its parts, since the BF does not interact with priming. More recently, McCormick et al. (2009) re-investigated frequency effects during masked priming, though without mentioning the results of the earlier studies reported here. They compared the effects of High SF, Low SF and pseudoword primes on target recognition, but contrary to Giraudo and Grainger (2000) they compared each priming effect on different targets (e.g., *brutal – brute* vs. *adorable – adore* vs. *agitatal – agitate*, respectively). They found facilitation effects on all three conditions relative to each of the three unrelated baselines (e.g., *verbal – brute, enviable – adore, corrodal – agitate*, respectively). In our view, the lack of orthographic controls that could separate formal from morphological effects constitutes a serious obstacle for the interpretation of their data, which thus only show that related primes facilitate target recognition. Furthermore, it is very surprising to see that despite an interpretation in favor of the decompositional hypothesis, these authors did not test BF effects, which should strongly determine decomposition and therefore the magnitude of priming effects.

Further evidence against the decompositional hypothesis comes from the studies conducted by Giraudo and Orihuela (2015) and Giraudo and Dal Maso (2016). These masked priming studies carried out for French and for Italian replicated the SF interference effect and revealed that while whole-word frequency speeds up lexical access, morphological priming effects are also modulated by the relative frequencies of the prime and the target. SF interference effects highlight the role of the whole word over its internal structure during the very early stages of word recognition, and indicate that whole-word characteristics are more important for morphological salience than those of the word’s subparts. However, this does not amount to claiming that morphological structure does not play a role. In our view, morphological salience emerges from relationships between whole word forms and their parts. The whole word guides lexical access, while morphological relationships are expressed by the links that cluster together word forms belonging to the same family or series (which cluster complex words according to the affix they share in common, e.g., *cleaner, hunter, biker*).

Finally, a set of studies that, in our view, contradict the mandatory decomposition hypothesis, use non-word primes involving transposed letters (TL) that disrupt the morpho-orthographic structure. Masked priming experiments have compared the effects of complex non-word primes with TL at a morpheme boundary (e.g., *painetr-paint*) to effects of primes with TL outside the morpheme boundary (e.g., *paniter-paint*). Although priming effects were obtained independently of the position of the TL (at the morpheme boundary or not), this has not lead researchers to call the decompositional approach into question (Perea and Carreiras, 2006; Rueckl and Rimzhim, 2011; Beyersmann et al., 2012, 2013; Luke and Christianson, 2012; Diependaele et al., 2013b).

We take issue with this interpretation, since if morphological decomposition governs access to word forms coded in the mental lexicon, non-word primes which cannot be parsed into distinct surface morphemes should not be able to induce priming. Since their surface morphological structure is hidden by the TL (e.g., *painetr*), no morphemic units should be activated and therefore no priming is expected. And even if a sublexical mechanism was able to recode letter position (as suggested by Diependaele et al. (2013a)), the position of the TL should interfere with morphological priming: letter-transposed primes with intact morphemic boundaries should be more effective for the recognition of their base (like *paniter – paint*) than those with disrupted morpheme boundaries (as in *painetr – paint*). Moreover, the mechanism of letter recoding must depend on a match between the prime and a whole-word representation coded at the word form level, which implies that the whole word guides access rather than its parts. In our view, rather than supporting decomposition, the data obtained with non-words constitute strong evidence in favor of holistic processing of the primes and, by extension, of all the stimuli, whatever their surface structure. We take the fact that words with jumbled letters can induce priming effects to provide sufficient grounds to reject the claim by Amenta and Crepaldi, according to which non-word effects cannot result from lexical activation. We interpret these data obtained with non-words in the opposite way: the pattern of systematic form-meaning correspondences that we call morphology (Bybee, 1988, 2001; Booij, 2010) has to be extended to all possible words.

Talking about morphological links implies taking into account another factor whose impact on complex word recognition has been demonstrated and replicated in various languages: morphological family size (i.e., the total number of words derived from the same morphological family; Bertram et al., 2000b; De Jong et al., 2000). It has been shown that complex words with many morphological relatives are processed faster than those with a small morphological family, suggesting that the locus of morphological effects is not exclusively the word to be processed, and that factors outside the word in question intervene in morphological processing. In the same line, Voga and Giraudo (2009) explored a novel variable, called the “pseudo-family size,” which is the opposite of the morphological family. The notion of pseudofamily size includes neighbors in the classic sense (i.e., members of the morphological family), but also all words sharing their stem with a given entry, even if what remains of the word once the stem is removed is not really an affix. Their working hypothesis was that pseudo-relatives should behave like competitors at the word level. This was tested in two masked inflectional priming experiments comparing two kinds of stimuli: verbs from large pseudo-families and verbs from small or non-existent pseudo-families. The first experiment studied the classic configuration, where the target is the easiest-to-activate member of a paradigm (e.g., *monté-monter* ‘climbed-climb,’ where the target *monter* has the highest SF in the family). By contrast, the second experiment took as targets less frequent inflected forms (e.g., *monté-montons* ‘climbed-we climb,’ where *montons* has a low SF within the family), thus reversing the typical design in which the target corresponds to the base, i.e., the member of the morphological family that already has the greatest residual activation because of its frequency. Under the conditions of the second experiment, only small pseudo-family-size verbs induce repetition and morphological priming, for both frequent and infrequent inflections, whereas large pseudo-family-size verbs fail to induce repetition or morphological priming. Moreover, inflectional priming for small pseudo-families verbs does not differ for the two types of primes, i.e., frequent or not frequent inflections. These data added new evidence to the view that both the lexical frequency of word-forms and relative frequencies between primes and targets influence morphological processing.

## The Salience of Whole Words in an Integrative Perspective

All the data presented in Section “Objections to the Decompositional Approach” can be interpreted in a way that is straightforwardly compatible with the holistic view. In our view, advocates of the decompositional view of word recognition have systematically confused two types of results: On the one hand, data obtained on the basis of complex words and non-words whose surface morphemes can be rapidly and easily extracted have been interpreted as supporting automatic morphological decomposition. On the other hand, obstacles to a perfect morphological segmentation have been attributed to the robustness of the decomposition mechanism.

Returning to the notion of morphological salience, this property as derived from the decompositional perspective is based only on the surface morphemic complexity of the stimuli and is opposed to another definition under which morphological salience emerges from form-meaning correlations. While the former reduces morphological to formal effects, the latter stresses the role of paradigmatic relationships between words without denying the role of morphemes during word recognition. Aronoff (2007) claims with respect to this issue that “[t]here is plenty of evidence, linguistic and psycholinguistic, for morphemes and roots and for morphological relatedness. But none of this evidence, pace Stokall and Marantz (2006), supports a purely morpheme-based theory over one that recognizes lexemes but also recognizes roots and morphemes as morphologically significant elements, albeit not as reliable Saussurean signs” (p. 813). In line with this statement, we recognize the existence of morphemes, but only as secondary and derivative units of description.

As mentioned above, the empirical data from the psycholinguistic literature so far have mostly been interpreted in favor of a decompositional view, which reduces morphological effects to formal effects. But if morphological salience only relates to the surface structure of words, this salience, which seems to guide the early stages of word recognition, cannot be called ‘morphological’ since morphological relationships are, by definition, pairings of form, and meaning (Blevins, 2014). On the other hand, numerous studies have shown that ‘morphological’ priming is distinct from mere formal relationships: *freeze* does not prime *free* while both *hunter-hunt* and *corner-corn* show facilitation effects. The relevant priming effect must therefore take place at a level which is more than formal, but less than morphological. However, this structural salience effect does not exclude a genuine morphological salience effect emerging from paradigmatic relationships between the word representations coded within the mental lexicon. In other words, we assume the co-existence of both morphological structure and whole-word salience effects, but while the former depends on quantitative factors such as the statistical occurrence of letter clusters (including those that correspond to morphemes), the later is determined by qualitative variables (e.g., the degree of semantic transparency) resulting from morphological relationships shared by words.

The present review has presented and discussed the factors which guide the processing and the lexical representation of morphologically complex words, and has given an overview of the highly controversial debate on possible interpretations of the results obtained so far. More specifically, we have shown that the issue of the relative prominence of the whole word and its morphological components has been overshadowed by the fact that psycholinguistic research has progressively focused on purely formal and superficial features of words, drawing researchers’ attention away from what morphology really is: systematic mappings between form and meaning. While we do not deny that formal features can play a role in word processing, an account of the general mechanisms of lexical access also needs to consider the perceptual and functional salience of lexical and morphological items.

We hold that results obtained on the basis of masked priming are in line with holistic models of lexical architecture or models in which morphology emerges from the systematic overlap between forms and meanings (Baayen et al., 2011). In such models, salience is not only a matter of internal structure, but also results from the organization of words in morphological families and series; as a consequence not only syntagmatic, but also paradigmatic relationships must be taken to contribute to morphological salience.

Certainly, the notion of salience refers primarily to formal aspects, because the perceptual body of the morpheme is necessarily the starting point of the processing mechanism. However, the notion of salience makes sense for complex word processing only if the form it refers to is associated with a meaning or function. Salience, in other words, is a property of the morpheme (i.e., a stable association of form and meaning), not simply of a phonetic or graphemic chain. We suggest that re-focusing attention on salience, rather than on purely formal aspects, could lead to more interesting interpretations of the data observed so far in the psycholinguistic literature.

## Author Contributions

HG: Psycholinguistic contribution; SD: Linguistic contribution.

## Conflict of Interest Statement

The authors declare that the research was conducted in the absence of any commercial or financial relationships that could be construed as a potential conflict of interest.

## Footnotes

1 These three masked priming paradigm studies associated ERP measures with RT recordings.
